# Functional Mechanisms of Health Behavior Change Techniques: A Conceptual Review

**DOI:** 10.3389/fpsyg.2022.725644

**Published:** 2022-03-18

**Authors:** Maren M. Michaelsen, Tobias Esch

**Affiliations:** Institute for Integrative Health Care and Health Promotion, Faculty of Health, Witten/Herdecke University, Witten, Germany

**Keywords:** behavior change techniques (BCTs), functional mechanisms, resources, conceptual framework, nudging, reward and motivation

## Abstract

**Background:**

Health behavior change is among the top recommendations for improving health of patients with lifestyle-related chronic diseases. An array of behavior change techniques (BCTs) have been developed to support behavior change initiation and maintenance. These BCTs often show limited success when they are not informed by theory, leading to a mismatch between the intention of the BCT and patients’ needs or expectations. Previous studies have identified a number of resources (domains) which patients may require to initiate and maintain health behavior change. Indeed, not yet well established is how BCTs address these resources, i.e., the functional mechanisms of BCTs.

**Purpose:**

Provide a theoretical framework of the functional mechanisms of BCTs for developing and implementing successful interventions for health behavior change.

**Methods:**

Conceptual review, including literature analysis and synthesis as well as conceptualization of a new model based on the synthesis.

**Results:**

Through the integration of dual-process models as well as reward and motivation proceeding, i.e., affective, emotional, or intuitive neurobiological cues, into the rational framework of rather linear cognitive or task-related decision progress, we categorize previously identified resources into three distinct sets: external, internal reflective, and internal affective resources. Based on this triad, we classify BCTs according to their functional mechanisms into facilitating (=providing external resources), boosting (=strengthening internal reflective resources), and nudging (=activating internal affective resources). Consequently, we present a simplified Behavior Change Resource Model (BCRM) that is centered on patients’ resources.

**Conclusion:**

The model can be applied to develop health behavior change interventions, which promote engagement and empowerment. Future studies should aim at testing the applicability and practicality of the BCRM.

## Introduction

Many chronic diseases can be improved, or prevented, through health behavior changes, such as increased physical activity, healthy eating, relaxation strategies, as well as adequate stress- and self-management, social support networks, a good work-life-balance, and the like (Knowler et al., 2002; Michalsen et al., 2005; Esch and Esch, 2016; Morris et al., 2019). Nowadays, health behavior modification is the key recommendation in most treatment guidelines for lifestyle-related chronic diseases and engaging the patient in his or her health has become a priority for practitioners, politicians, and stakeholders (Esch, 2018). For example, a survey among the NEJM Catalyst Insights Council reveals that 90% of its members hold patients responsible for weight loss (Volpp et al., 2008), primary care professionals recognize patients as central to disease management (Jallinoja et al., 2007), and plenty of research exists on the relevance of autonomy of patients with chronic diseases (e.g., Bodenheimer et al., 2002; Kennedy et al., 2007; Uysal et al., 2017). However, most individuals have difficulties in initiating, and more so maintaining, health behavior changes. This is no surprise as humans are exposed to a series of temptations in everyday life that promise immediate pleasure. These often come into conflict with, and can ultimately undermine, long-term behavior goals, which promise future health improvements (Loewenstein, 1996; Stroebe et al., 2008, 2013; Hall and Fong, 2015).

Despite being so essential to enhance health, patient support to implement health behavior change recommendations into their everyday life is rarely covered by healthcare systems around the world (Chauhan et al., 2017; Grabovac et al., 2019). At the same time, a large amount of interventions has been developed and evaluated by research teams and private enterprises. Among those interventions are nutritional or psychological counseling (e.g., Ball et al., 2013), assisted walking groups (e.g., Kassavou et al., 2013), financial incentives (e.g., Lee et al., 2019), wearables (e.g., Piwek et al., 2016), and other digital innovations (e.g., Priesterroth et al., 2019), as well as reminders (e.g., Orr and King, 2015) among various forms of nudging, i.e., arranging decision-making contexts in ways that promote a certain behavior (e.g., Hansen and Jespersen, 2013). Despite a rapid growth in intervention implementation, most of these interventions are only successful in the short-run, and often cannot demonstrate a significant improvement in the medium- and long-run (e.g., Marteau et al., 2012; International Diabetes Federation [IDF], 2013; Ulrich et al., 2016; Sainsbury et al., 2019). The majority of existing interventions also is too costly to be carried out over longer periods or be provided for a large number of individuals (Forster et al., 2011). Over and above, interventions may have produced counter-effects later on. For example, financial incentives for weight loss have been shown to be effective up until the financial reward has been received, but tend to produce weight gain in the weeks following payoff (Paul-Ebhohimhen and Avenell, 2008). Explaining such findings is not straightforward, and the need for both effective and cost-effective health behavior change interventions calls for a more thorough understanding of the functional mechanisms of behavior change techniques (BCTs). In fact, in a scoping review on nudging interventions, the authors found that three out of four studies solely aimed at demonstrating effectiveness of the intervention, whilst only one fourth paid attention to the hypothesized “working mechanisms” of their effectiveness (Szaszi et al., 2018). These working mechanisms often refer to the links between BCTs and the targeted resources or domains, i.e., which resource is addressed by which BCT (Carey et al., 2019). A current research gap is the understanding of the functional mechanisms of BCTs, i.e., how BCTs address resources. Filling this gap would allow a more specified theoretical understanding and a more precise development of intervention components that are more likely to prove effective in the long-run.

In order to provide such understanding, in this conceptual review, we identify key constructs of BCTs and offer a conceptual framework, which is rich in details and at the same time simple but precise in terms of its abstraction, thereby generating promising content for future research. We do so by integrating recent developments in neuroscience, especially dual-process models, as well as accounts of motivation and reward proceedings, which may help to elucidate the functional mechanisms of BCTs.

Our elaboration is based on four steps. First, we define BCTs and highlight their role within a general behavior change process that requires different resources. Second, we synthesize the various resources (domains, determinants) suggested in a number of published theoretical constructs through the incorporation of neuroscientific knowledge of motivation and reward proceedings, and dual-process models. Based on this synthesis, third, we categorize BCTs corresponding to their functional mechanisms, i.e., how they target these resources, into three types. Fourth, this allows presenting a novel framework of behavior change that is centered on a patient’s resources; the Behavior Change Resource Model (BCRM).

By focusing on resources rather than barriers, faults and shortcomings, we stress positive aspects of human functioning and flourishing on multiple dimensions (cf. Seligman and Csikszentmihalyi, 2000). This provides a multi-facetted view on individual circumstances, abilities and actions that may interact positively with other aspects of life, over and above the behavior that is in focus of the BCT (see vantage resources; e.g., van Cappellen et al., 2018). Finally, we discuss limitations, relevance, as well as implications of the model and place each identified type of BCT into an empowerment framework in order to guide future intervention development and implementation.

The article is structured as follows; the relevance, definition, and basic goals of BCTs are described in the beginning (next chapter). The third chapter defines behavior change resources, explains the relevance of dual-process models for categorizing behavior change resources, and offers a threefold classification of behavior change resources, i.e., internal reflective, internal affective and external resources. Based on this classification, in the following chapter, BCTs are clustered into three sets: Facilitating, Boosting and Nudging techniques. This elaboration is presented in form of a new model, the BCRM in the section that follows. The relevance of the model, its normative implications, its potentials for empowerment and autonomy are discussed in Chapter “Discussion,” which also includes an application guide for practitioners in private, public, corporate and clinical settings. The limitations of this review and suggestions for future research are discussed in Chapter “Limitations and Future Research,” and the last chapter summarizes the review and offers a conclusion.

## Relevance, Definition, and Goals of Behavior Change Techniques

Research on health behavior change has generated a number of multi-stage health behavior change theories that aim at explaining the various steps an individual has to undergo in order to take up and habituate a new health behavior. Based on established theories, Michaelsen and Esch (2021) have developed and defined a seven-stage behavior change process. In this process, individuals may experience the stages unawareness, awareness, contemplation, planning, initiation, continued action, and maintenance. These stages are categorized into three phases of engagement, namely, non-engagement, motivational engagement, and executive engagement, in which individuals’ actions are driven by different types of motivational and endogenous reward processes. At each stage, various resources (e.g., knowledge, behavioral regulation, or reinforcement) are required to progress from one stage to another. A number of well-established social-psychological theories have provided suggestions and explanations of the determinants of health behavior change initiation (e.g., Bandura, 1989; Gollwitzer, 1999; Ryan and Deci, 2000) or maintenance (e.g., Prochaska et al., 1988; Weinstein and Sandman, 1992; Rothman et al., 2004; Schwarzer and Luszczynska, 2008), which we do not elaborate here in detail due to their extensive discussion throughout the health behavior change literature. These theories have been applied to develop BCTs in various ways, e.g., by integrating social interactions (see Luszczynska and Schwarzer, 2020, for an overview of BCTs) based on Bandura’s (1989) social cognitive theory, or by helping patients to generate implementation intentions (see Bélanger-Gravel et al., 2013 for a meta-analysis of BCTs) based on Gollwitzer’s (1999) implementation intention theory.

According to Carey et al. (2019), a BCT is defined as a replicable component of an intervention that aims to alter causal processes that regulate behavior of a target population. These BCTs are either augmenting factors that promote behavior change or moderating factors that inhibit behavior change (Carey et al., 2019). Traditionally, health behavior change interventions have tended to focus on making new information available or providing other incentives (e.g., financial or legal) that change behavior or attitudes toward one’s own behavior in a relatively generic manner (Cecchini et al., 2010). Modern BCTs frequently integrate content that is more relevant to the individual, i.e., BCTs are often tailored to the individual’s or specific target population’s needs. Furthermore, modern interventions more often actively engage participants. Such BCTs often build upon cognitive resources, such as goal setting (development of goals and steps to achieve them), action planning (development of a plan that contains certain actions) and self-monitoring (self-observation in order to regulate behavior), in which participants reflect upon their own (future) behavior. Another increasingly applied set of BCTs integrates social support, e.g., walking groups (e.g., Kassavou et al., 2013), and more and more digital BCTs (eHealth and mHealth; see, e.g., Kebede et al., 2017) have been and are being developed, implemented and evaluated. Meta-analyses have shown that interventions that have participant-relevant content and trigger participant engagement tend to be more effective (Noar et al., 2007; Lustria et al., 2013).

In an extensive review, Carey et al. (2019) have listed BCTs applied in 227 interventions, which aimed at changing health behavior. They have also linked these BCTs to their mechanisms of action, i.e., the resources through which they affect the behavior of interest. As an example, they suggest the BCT ‘instruction on how to perform the behavior’ to be linked to the resources knowledge and skills, and the BCT ‘goal setting’ to behavioral regulation (Carey et al., 2019, p. 700). Their results are comprehensive in that they show which BCTs target which resources (which they name mechanisms of actions), but neglect how they do so. Going beyond the provided links between BCTs and targeted resources, i.e., through which functional mechanisms (e.g., neurobiological processes) the BCTs affect the resources provides an even better understanding of BCTs. Hence, the present study elaborates upon Carey et al.’s (2019) review, and provides an understanding of the functional mechanisms of BCTs. This is done by, e.g., considering recent neuroscientific evidence on affective-emotional cues, i.e., motivational and reward processes, and their change over time. Doing so, we aim for an even better comprehension and development of BCTs.

## Synthesizing Behavior Change Resources

### Defining Behavior Change Resources

Previous research has used a number of terms for behavior change resources, such as domains, determinants, augmenting factors, or mechanisms of actions (e.g., Strack and Deutsch, 2004; Cane et al., 2012; Carey et al., 2019). Here, we use the term behavior change resources (in short: resources), which we define as factors of an individual that can affect behavior; factors related to an individual’s external environment (socio-environmental resources) and factors of an individual’s internal environment or state (bio-psychological resources), that can either be changeable or non-changeable. The resources discussed in the literature are commonly–often without being explicitly stated as such–changeable, although, we argue, there are resources that can affect behavior that are non-changeable, such as weather conditions. Hence, it is necessary to distinguish between non-changeable resources and changeable resources. Changeable resources are usually acquired and amendable. These, again, are comprised of socio-environmental, biological, and psychological states of the individual. Changeable socio-environmental resources are acquired, changeable conditions prevailing outside of the individual’s body or mind, such as living conditions, including tangible resources or social support. Changeable biological resources are amendable conditions referring to the individual’s bodily state, such as physical health, including fitness, metabolic status, weight, or cardiovascular health. Finally, changeable psychological resources are amendable qualities, which are built, for example, through information processes and self-regulatory capacities within the brain.

Non-changeable resources are defined as immutable factors of the individual. These resources refer to the individual’s innate biological and psychological qualities or socio-environmental circumstances, such as personality traits, age, height as well as climate and culture. BCTs can focus on developing strategies to circumvent or take advantage of non-changeable factors. For example, for aged inviduals, who are incapable of long walks (which could prevent health problems due to sedentary behavior), a suitable BCT could be, e.g., a seated yoga intervention. The resource targeted in this case is skills or needs–which are changeable–rather than the non-changeable resource “age.” As such, we define that BCTs can only target changeable resources, and we only focus on those in the elaboration that follows.

A number of theoretical constructs aiming at explaining the roles of health behavior change resources have been published over decades. These publications provide discussions of either single resources or models and frameworks, which suggest various resources relevant for health behavior change (e.g., Bandura, 1989; Gollwitzer, 1999; Ryan and Deci, 2000; Michie et al., 2005; Fishbein and Ajzen, 2010; de Bruin et al., 2012). Resources have been summarized, e.g., into an encompassing framework, the “Theoretical Domains Framework” (TDF) by Michie et al. (2005) and Cane et al. (2012). The TDF has been developed by a large number of experts from psychological theory, health services research, and health psychology. It consists of 14 theoretical domains (Cane et al., 2012), which are listed in Table 1. We regard the TDF a rather comprehensive list of changeable resources and use it as an exemplary register to advance our following elaborations. Details about each of these resources can be found in Cane et al. (2012). The previously mentioned review on BCTs by Carey et al. (2019) has also built upon the TDF and links BCTs to the domains established therein.

**TABLE 1:** Fourteen Behavior change resources (domains), as defined by Cane et al. (2012).

Knowledge
Social/Professional Role and Identity
Optimism
Reinforcement
Goals
Environmental Context and Resources
Emotions
Skills
Beliefs about Capabilities
Beliefs about Consequences
Intentions
Memory, Attention, and Decision Processes
Social Influences
Behavioral Regulation

### Dual-Process Models

Previous social-psychological models on health behavior change have mostly focused on cognition and intentional deliberation (Kwasnicka et al., 2016). Even more recent models, which not only aim at explaining initiation but also maintenance of new behavior, mainly discuss the role of cognitive resources. In a review of health behavior change maintenance theories, Kwasnicka et al. (2016) found that only ten of the 100 behavior change maintenance theories they analyzed, point out the relevance of automatic responses to cues or stimuli in addition to cognitive, or reflective aspects of behavior. van Cappellen et al. (2018) pose that it is a limitation to existing theories that they emphasize cognitive resources. Furthermore, in a meta-analysis of BCTs’ effectiveness, Webb and Sheeran (2006) found that medium-to-large changes in intention produces only a small-to-medium effect (change) in behavior (*d* = 0.36). Taking into account also Sheeran et al.’s (2014) result that a large change in risk perception has only a small effect on behavior (*d* = 0.23), Sheeran et al. (2014, p. 460) conclude that “changing conscious thought does not, it seems, guarantee health behavior change.” A fully comprehensive definition of behavior provided in a recent meta-analysis of interventions to maintain behavior change further highlights the relevance of non-cognitive recourses as relevant factors for behavior change; here, behaviors are defined as “[…] physical events that occur in the body and are controlled by the brain” (Kwasnicka et al., 2016, p. 280). In contrast to social-psychological theories, which have not taken into account the role of the brain–or, more precisely, neurobiological processes in the brain–in determining behavior, the relevance of such processes has been discussed in the more separate strand of literature of dual-process models (e.g., Strack and Deutsch, 2004; Evans, 2010; Sheeran et al., 2013). This type of models provides valuable information for understanding the functional mechanisms of behavior change techniques.

Dual-process models of decision-making differentiate between reflective (cognitive, conscious) and affective (impulsive, intuitive, automatic) precursors of behavior through the interplay of two regulatory systems in the brain (Chaiken, 1980; Petty and Cacioppo, 2012). The reflective system is based on conscious deliberation and control which is subjectively effortful. It is based on rules of language and logic, and draws upon an individual’s knowledge of probabilities and values. Its key processes are intending and reasoning which can be accessed intentionally. The reflective process is relatively slow (Strack and Deutsch, 2004; Sheeran et al., 2013). It tends to override a quicker, more effortless, automatic system. This draws upon the store of associations acquired through experiences, i.e., it responds to habits and impulses. Strack and Deutsch (2004) describe the system as an important impulsive process that generates activation, with perceptual inputs activating elements in associative memory, which in turn activate other related elements. This type of information processing is fast and occurs outside of consciousness (Strack and Deutsch, 2004; Evans, 2010; Sheeran et al., 2013).

The roles of automatic and non-cognitive processes in behavior change have also been analyzed by Michaelsen and Esch (2021). In addition to information processing, which are focus of previously discussed dual-process models, in their integrative review, they take into account the neurobiological processes of motivation and reward proceedings, which also allow distinguishing between stimuli-driven behavior and goal-directed behavior. Stimuli, or cues, originate from internal or external sources and can activate either of the three types of motivation (approach, aversion, or assertive motivation) *via* reward expectations, i.e., the desire to experience a positive affect (Berridge, 2012, 2018; Michaelsen and Esch, 2021). The activation of motivational salience (i.e., the process that motivates behavior) through stimuli can occur unnoticed by the individual and lead to a behavioral response without reflection and/or intention (Ryan and Deci, 2000; Carver, 2009; Kruglanski et al., 2014; Berridge, 2018). Such motivationally unconscious states and processes are, we argue, essentially affective states and processes, as has also been pointed out by Billon (2011).

According to Michaelsen and Esch (2021), stimuli convert into goals when a reflective process occurred, i.e., the individual is aware of the stimulus and the motivational impulses resulting from it. Hence, behavior can be stimulus-driven or goal-directed. Furthermore, the fact that stimuli can result in action and engagement even without the individual being aware of it, stresses the relevance of non-cognitive, affective resources that can be activated through eliciting (non-conscious) reward expectations. Recent work has also demonstrated that the formation of goals can also be influenced through motivational stimuli that remain unnoticed (Bargh et al., 2001; Custers and Aarts, 2010). In particular, Aarts et al. (2008) have suggested an affective/motivational account of goal-pursuit in which motivational cues, e.g., nudges, strengthen goal activation and maintenance, even when this occurs outside of an individual’s awareness (Chiew and Braver, 2011). Autonomous goal formation (i.e., the formation of goals without the individual intentionally forming them) occurs, for example, because other peoples’ goals can be “contagious,” for example, through social comparison (Custers and Aarts, 2005, 2010). Heatherton and Wagner (2011) take into account motivational cues in their balance model of self-regulation, which suggests that self-regulatory failure occurs whenever top-down control from the prefrontal cortex over subcortical regions is unsuccessful. This may occur when reward and emotion is not balanced by the prefrontal cortex in order to resist strong impulses. Another neuroscientific account of the dual-process view is the elaboration on neural systems of habit (automatic) and goal-directed action control (cognitive) by Wood and Rünger (2016). They point out that habits both strengthen through automatic reward-learning processes, but can also, but slowly, be changed through deliberate goal pursuit or by changing cues in performance environments.

Two-systems models remain controversial (Evans, 2009; Keren and Schul, 2009), and claims for more systems or other explanations for behavior or decision-making exist (e.g., Foxall, 2016). However, through the application of dual-process models to health behavior (Hofmann et al., 2008; Friese et al., 2011) and decision-making (Hall and Fong, 2015), as well as psychological syndromes, including depression (Beevers, 2005; Turel and Bechara, 2016), dual-process models have contributed to an improved understanding of health phenomena. In addition, dual-process models already found applications in a number of settings, for example, in predicting sedentary behavior (Maher and Conroy, 2016; Arnautovska et al., 2017; Phipps et al., 2021). We believe the integration of such models also provides ground for a better understanding of the functional mechanisms of BCTs.

### Clustering Behavior Change Resources

By considering the distinction between reflective and affective processes in the brain and how they relate to behavior as well as the differentiation between changeable and non-changeable resources, we can establish a categorization of resources according to how they are being accessed or generated in the brain. We suggest that the aforementioned internal (bio-psychological) resources can be distinguished into reflective resources and affective resources. The former are those, which are generated, accessed or improved through effort and conscious deliberation, such as goals and behavioral regulation. Affective resources, in contrast, can quickly be activated through stimuli without intentional effort. Among these resources are emotions (affects) and their reinforcement. Next to these internal reflective and affective resources, we suggest an additional category, namely, external resources (socio-environmental), such as environmental context and material resources, as already defined in the preceding chapter.

**FIGURE 1:**
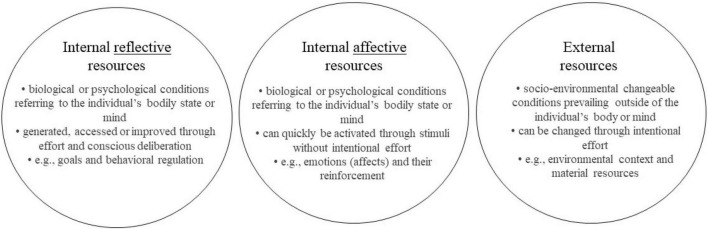
Behavior change resource clusters.

In summary, we believe it is worthwhile to distinguish three critical dimensions of resources relevant to behavior change: external, internal reflective, and internal affective (see Figure 1). This triad of resources forms the basis to elaborate further on the functional mechanisms of BCTs.

## Categorizing Behavior Change Techniques by Their Functional Mechanisms

The reduction of resource types into three categories (external, internal reflective, and internal affective) allows constructing three types of BCTs according to how they address these three types of resources, meaning their causal pathways through which they affect behavior. We name these types of BCTs facilitating, boosting and nudging, and explain their functional mechanisms in the following.

### Facilitating

Behavior change techniques can provide external resources, i.e., those that are outside of the individual’s internal processes. By providing these external resources, they facilitate the new behavior. In line with the distinction of resources made above, a BCT can only be defined as facilitation when external resourced are provided. Referring to the TDF (Michie et al., 2005; Cane et al., 2012), these resources are categorized for example within “environmental context and resources” as well as “social influences.” Thus, facilitating provides context, which enables the implementation of the desired behavior. Depending on the nature of the resource addressed, the resource can be provided by the individual, another person, or an organization. Examples for interventions of this type are adding objects to the physical environment, e.g., by offering healthy dishes at workplace canteens (Geaney et al., 2013), financial gifts (Petry et al., 2013), restructuring the physical environment, e.g., by developing public fitness trails (Cohen et al., 2012), as well as providing social support, for example, through the organization of assisted walking groups (Kassavou et al., 2013).

The behavior change initiated through facilitating BCTs can, by definition, only last as long as the provision is sustained. However, has an individual generated a certain degree of routine or habit of a particular new behavior, i.e., when the motivation to continue is specifically high, the termination of the BCT could lead to a substituting (similar) behavior that is implementable yet independent of the initial BCT. For example, the ending of an organized walking group could result in participants continuing to walk alone or self-organize their walking groups.

### Boosting

Reflective resources can be addressed by incorporating cognitive involvement of the individual. These resources can be modified using boosts, i.e., enjoyable tasks that will build up or strengthen internal reflective resources that can support behavior change. According to Hertwig and Grüne-Yanoff (2017), boosts foster competencies through changes in skills, knowledge or decision-making tools. Referring to Cane et al.’s (2012) classification of domains, we can add “beliefs about capabilities,” “beliefs about consequences,” “intentions,” “goals,” “memory, attention and decision processes,” and “behavioral regulation”’ to the list of resources that can increase the likelihood of reflective behavior change. Boosting interventions are typically non-regulatory and non-monetary (Hertwig and Grüne-Yanoff, 2017), such as self-monitoring of behavior and outcomes of behavior through, e.g., diary keeping, or mindfulness training (Shomaker et al., 2019) to enhance attention and awareness, as well as health education (Gigerenzer et al., 2007), and nutritional counseling (Ball et al., 2013) to increase beliefs about consequences, including many others (see, e.g., Grüne-Yanoff and Hertwig, 2016).

When sufficiently strengthened, the resource increases the readiness to change and will allow the intentional implementation of the desired behavior. The generated effects should persist once the successful intervention is removed because, once in place, those competencies are assumed to be stable over time (Hertwig and Grüne-Yanoff, 2017).

### Nudging

Nudges are interventions that steer people in a particular direction through changing aspects of the choice architecture while preserving their freedom of choice (e.g., Thaler and Sunstein, 2008; Alemanno and Sibony, 2015; Halpern and Sanders, 2016). Through intentionally applying stimuli, cues, or triggers in an environment to drive behavior of individuals or groups, nudging activates affective components of decision-making, which make the desired behavior motivationally more attractive–with freedom of choice still persisting–and its implementation playful and intrinsically rewarding (Michaelsen and Esch, 2021). In other words, nudges increase motivational salience and hence lead to a temporary increase in the (non-conscious) desire to obtain an associated reward. In this sense, nudges aim to alter behavior without straining cognitive resources but by strategically using automatic processes. In this way, by activating affective, automatic, or non-conscious resources, nudging can compensate for the lack of external or reflective resources that would be needed to induce behavioral change. Thus, nudging interventions (BCTs) do not depend on cognitive skills or the provision of external resources to be effective (van Gestel et al., 2020). Preliminary evidence supports this claim (Brunner, 2013; Hunter et al., 2018).

Examples of nudging interventions are specific presentation styles of food (Bucher et al., 2016; Broers et al., 2017; van Gestel et al., 2020), reminders or reinforcement learning strategies (Orr and King, 2015; Yom-Tov et al., 2017), lotteries (Volpp et al., 2008), and point systems (gamification) (Priesterroth et al., 2019); the latter is a type of reinforcement strategy (Kahneman and Tversky, 1979; Kahneman, 2011) which increases reward expectations. The rationale here is that the intensity of an expected reward determines how likely an individual is to remember and repeat it (Esch and Stefano, 2004).

Nudges can be applied by individuals themselves, as well as by others (organizations, governments, therapists, family, and friends). Whilst the latter may be the predominant ways to apply nudges, a set of “self-nudges” already exist. Considering the findings of fruit placement experiments (Wansink et al., 2011; Hansen and Jespersen, 2013), for example, an individual is likely to eat more fruit when a bowl with fresh fruit is placed where one regularly passes by and looks at routinely, compared to when fruit is placed in a more hidden space. This type of priming, as one form of nudging, is likely to increase the awareness of fruit and thereby the probability of grapping and eating more of it. The proximity nudge, in which a particular type of food is placed closer than other choices, has been shown to work in various studies (van Gestel et al., 2020). These types of nudges might work also when the individual herself arranges the food placement.

In sum, nudging interventions use resources without requiring effortful reflection. It follows that the effects, as a behavioral reaction to the increased motivational salience, is likely to last only as long as the nudge is applied. As an example, a multi-component intervention with point-of-decision prompts to foster stair use in a university dormitory showed no effects after the prompts had been removed (Howie and Young, 2011). However, because of learning processes as one component of motivation and reward proceedings, frequent repetition of nudged behavior may lead to a new habit (automation) that is eventually independent of the nudge, i.e., it would then persist even when the nudge has been removed from the individual’s surrounding (Verplanken and Aarts, 1999; Lieberoth et al., 2018).

## Presenting the Behavior Change Resource Model

**FIGURE 2:**
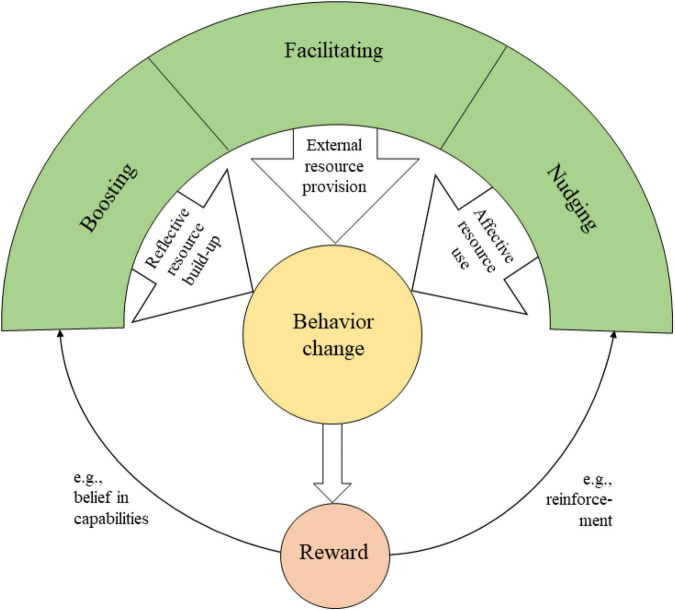
Behavior change resource model. Definitions of Boosting, Facilitating, and Nudging techniques and how the resulting behavior change affects reward processes.

The above classification of BCTs based on the functional mechanisms encompasses all existing BCTs, i.e., any BCT (e.g., all those listed in Michie et al., 2013) can be assigned to facilitating, boosting or nudging BCTs, or potentially to more than one, depending on the intention of the implementation of the BCT. Providing social support, for example, is a facilitating BCT; giving instructions on how to perform the behavior can be classified as a boosting BCT; and installing cues and prompts is a nudging BCTs. Based on the categorization of BCTs according to how they target health behavior change resources, we define resource-driven behavior change as a process that makes a desired behavior more likely by focusing on the resources necessary for the particular behavioral change to occur. Resource-driven behavior change results from one or a combination of the three types of BCTs that provide external resources (facilitating), build up internal reflective resources (boosting) or activate internal affective resources (nudging). If sufficiently strong, the applied BCT leads to the initiation or maintenance of a new behavior, and a reward in form of positive affect is generated. This reward can then serve as a cue or stimulus itself and subsequently improve resources (vantage resources; van Cappellen et al., 2018). For example, a reward experienced in form of a positive affect can act as reinforcement and thereby be a nudge itself, as the experience of the pleasant affect is critical to forecasting subsequent behavioral engagement. Accordingly, the reward experienced from successful implementation or repetition of the desired behavior can also, for example, strengthen the belief in one’s own capabilities, thereby act as a boosting strategy. Thus, the functional mechanisms of BCTs cannot be understood to be independent of, but interlinked with, neurobiological motivation and reward proceedings. By acknowledging these multidirectional causal relations, we herewith present a novel framework for understanding the functional mechanisms of BCTs: the *Behavior Change Resource Model* (BCRM). This model is depicted in Figure 2.

## Discussion

### Relevance of the Behavior Change Resource Model

By developing the BCRM, we aim at providing a source for a better understanding of the mechanisms through which BCTs lead to behavior change. We argue that BCTs target individuals’ resources in particular ways, namely, by either providing external resources (facilitating), building up or strengthening internal reflective resources (boosting), or by activating affective resources (nudging). This clustering of BCTs is made possible through the integration of motivation and reward mechanisms, which can be noticed and therefore consciously reflected by the individual, or can remain unnoticed by the individual and thereby lead to automated behavior. This argumentation is in line with dual-process models and the differentiation between stimuli-driven and goal-directed behavior.

The basis of our argumentation is our belief that individuals are diverse in terms of their resource endowments and resource needs. In contrast to other frameworks of health behavior change, we have taken this aspect as the cornerstone of our framework. It allows identification or development of appropriate BCTs according to the target populations’ or individual’s resource endowments and requirements. Such BCTs are most promising as they engage the patient in his or her health and ultimately lead to new health habits. How to identify the resources a particular patient or group requires is still subject to future research. Preliminary suggestions, such as interview questions posed in Rollnick and Miller (1995) and Michie et al. (2005) exist. Based on the identification of the resource(s) that are relevant to the individual, an intervention can be planned that consists of the BCT or set of BCTs that target these resources. Thereby, any BCTs (e.g., those listed in Michie et al., 2013) can potentially be combined.

### Normative Implications

In defining the three types of BCTs, we partly deviate from previous studies. First, facilitating BCTs have not been discussed or defined as a single category so far. Therefore, we created a generally new family of BCTs that are important to take into consideration when developing behavior change interventions. The provision of many external resources, such as access to facilities and the fulfillment of basic needs (see Maslow, 1943; Taormina and Gao, 2013), is a policy relevant issue *per se* and its implementation requires political measures. Second, our definition of boosts is in line with the one for long-term boosts discussed by Sunstein (2015) and Hertwig and Grüne-Yanoff (2017), but excludes interventions that target the individual’s environment (Grüne-Yanoff and Hertwig, 2016). The latter we here define as facilitating BCTs, as their functional mechanism is different to the functional mechanism of boosts. Third, our nudging definition is an extension to previous definitions and at the same time a simplification. Whilst we do not argue against the common definitions of nudges (Rebonato, 2012; Hansen, 2016), we see differences in the reasons for applying them and their causal pathways. In Sunstein (2015), the author introduced educative nudges, such as information, warnings, and reminders. These are seen as short-term boosts by Hertwig and Grüne-Yanoff (2017), which is more in line with our view. Hertwig and Grüne-Yanoff (2017) have argued that educative nudges present corrections for a problem and demand a minimum of motivation and cognitive effort–which is supposed to be irrelevant for classical nudges and fits more the definition of short-term boosts. This is based on the argument that the nudging approach’s starting position is that individuals exhibit deficits in decision-making competence (Fiske and Taylor, 1991; Kahneman, 2011), and that those are pervasive and difficult to alter (e.g., Grüne-Yanoff and Hertwig, 2016).

We extend this view through the argument that nudges also target resources. This is in contrast to Hertwig and Grüne-Yanoff (2017), for example, who claim that nudging interventions target behavior and boosting interventions foster competencies. At the same time, Hertwig and Grüne-Yanoff (2017) note that learning incentivized through nudges may lead to routines of new behavior, thereby affecting cognitive and motivational processes. In other words, repeated nudges themselves may strengthen resources through the generation of reward experienced through the execution of the new behavior. That rewards experienced in terms of positive affect can further strengthen other behavior change resources has indeed already been shown by van Cappellen et al. (2018) and Fredrickson et al. (2020). In a sense, Hertwig and Grüne-Yanoff (2017) generate a contradictory argument to their claim that nudges only target behavior and boosts only foster competencies. We suggest that all three types target resources in order to change behavior–but do so in different ways. Thereby, we present the mechanisms of how BCTs affect behavior in a more consistent way. Nevertheless, we build upon the common view that nudges take advantage of effortless and automatic processes involved in decision-making.

Basis for deciding which BCT to choose in order to alter a particular behavior are, commonly, efficiency, effectiveness, and welfare, especially for policy interventions. Nudges have often been highlighted to be best combining cost-efficiency and large-scale impact, i.e., maximum net benefits, as compared to public education or traditional economic policy (e.g., Weber and Johnson, 2009, p. 75). This is, according to Hertwig and Grüne-Yanoff (2017), a misconception.

Efficiency, effectiveness, and welfare are nevertheless relevant arguments. Next to these decision points of intervention development and planning, the normative implications of BCTs are of great importance. We therefore discuss the three types of BCTs established herein according to their potential to empower individuals.

### Empowerment

Empowerment is a term that has been applied in different contexts, such as healthcare, employment, and learning (Novak, 2002; Zhang and Bartol, 2010). Traditional approaches to patient care tend to ignore individual patient preferences (The Lancet, 2012). Including patient preferences, however, could promote patient empowerment. A consensus about what patient empowerment actually is, still remains to be found, however. Anderson and Funnell (2010) provide a definition we find most suitable in the context of behavior change: “Empowerment is a measurable increase in the patients’ ability to make autonomous, informed decisions” (p. 278). This implies that empowerment can both be a process or an outcome of an intervention, i.e., the intervention results in “empowered behavior” (Fumagalli et al., 2015, p. 387). In this light, BCTs can be viewed as empowering processes to become engaged in new, healthier behaviors. Fumagalli et al.’s (2015) review on the definition of empowerment also includes the concept of motivation: Empowering individuals to make decisions is equivalent to activating motivational processes. Hence, we define the basic dimensions of empowerment autonomy, transparency, and motivation. In the following, we briefly discuss the degree of empowerment potentially resulting from the three types of BCTs.

We suggest that facilitating, as constructed here, is the most direct way to empowerment, as the provision of external resources leads to a reduction in perpetuated impossibilities to conduct a behavior. Once provided, the individual can autonomously conduct a behavior. Facilitating BCTs are transparent and likely to increase motivation. Therefore, the empowerment process through facilitation, we suggest, is a direct empowerment path: Facilitating is directly enabling behavioral change.

In boosting approaches, like cognitive behavioral therapy, the participating individual is assumed to be reflectively involved in the activity; these approaches often require time, effort, cognitive capacity, and motivation from both the provider (e.g., teacher or therapist) and the participating individual. Thereby, boosts pursue the aim of preserving personal agency and enabling individuals to practice that agency (Adams et al., 2016; Hertwig and Grüne-Yanoff, 2017). Boosting approaches are necessarily transparent, as the individual can choose to not engage in them. They also increase individual’s autonomy to execute a desired behavior over time, and the individual/patient might profit from the newly built up resource in other areas or aspects of life (van Cappellen et al., 2018). Hence, we suggest boosting BCTs generate a self-directed empowerment processes.

Nudging, in comparison, does require neither extra time nor effort for resources to be built up. Instead, as nudging circumvents the lack or weakness of resources, it can still be useful when other resources are scarce or impossible to be provided. By activating affective, automatic decision-making processes, behavior change is likely to be prompt and effortless. Nudging BCTs, because of their potential to operate through the quick and non-conscious system, we suggest, neither leads to direct nor self-directed empowerment. Rather, we believe nudging exhibits indirect empowerment. Against the argument occasionally posited that nudging undermines self-determination, it may actually lead to empowerment, if individuals become engaged in their desired change process. Even if the presence of motivational engagement is subliminal, nudging can still generate executive engagement and thereby lead to progress along the change process. This is because executing a desired behavior without reflective effort or focused attention will induce an endogenous reward no matter how (explicitly/consciously) motivationally involved the individual has been beforehand. Subsequently, this reward is likely to motivate for further repetition. In sum, nudging has the potential to reinforce endogenous reward experience when the new behavior is repeated and, hence, to increase positive affect experienced through the new behavior. Only this latter aspect indicates the preservation of autonomy, whilst short-term or single nudges could undermine autonomy when the individual has not agreed to be supported through nudges. It would be interesting to examine whether and how affective states stimulated through nudges are experienced by the decision-maker.

### Autonomy vs. Manipulation

The potential lack of transparency of being nudged is the reason for a debate about whether nudging is a form of libertarian paternalism (Thaler and Sunstein, 2008; Blumenthal-Barby and Burroughs, 2012; White, 2013). Sunstein (2015) argues that nudges can be justified on grounds of autonomy, dignity and welfare, while others discuss whether certain forms of nudges are manipulations, and in which cases they might be legitimate (Nys and Engelen, 2017). Yet, in which cases manipulations could be justified, is exemplarily stated by Blumenthal-Barby and Burroughs (2012, p. 5): “One can bypass a person’s reasoning capabilities for good reasons (e.g., the person’s reasoning powers are impaired) and for good ends (e.g., to prevent the person from harming themselves).” Other authors (e.g., Cohen, 2013; Thaler, 2018) argue that, from a policy perspective, risks and benefits of manipulating someone to change their behavior using nudging techniques have to be weighed against reasons other BCTs, or not intervening at all. For example, in the case when a lack of behavioral regulation is the (missing) resource preventing an individual from reaching his/her stated desired behavioral goal, nudges can still help to circumvent this weakness and help to satisfy individual preferences spontaneously (Sunstein, 2018). A boosting BCT, such as self-regulation training, which is time and effort intensive for the individual (and providers), could be an additional option pursued simultaneously in order to supersede nudges in the long-run. From our point of view, the choice of BCTs, especially nudges, should be made very carefully and be in line with the target group’s or individual’s goals.

### Guide for Practitioners

In order to provide guidelines on how to use the model, its specifications and implications discussed so far are summarized in Table 2. For each type of BCT defined above, the table contains a comprised definition, and the resources targeted. The resources listed here are those summarized in the TDF (Cane et al., 2012) as discussed in Chapter “Defining Behavior Change Resources.” Some of these resource examples could be assigned to more than one functional mechanism, as they can be intepreted as both, e.g., reflective and affective (such as attention) or can be improved (strengthened/activated) through several types of interventions (such as financial gifts, as discussed above). The table also provides “umbrella” BCT examples from to Carey et al. (2019) for each functional mechanism including practical intervention examples. A further column of the table contains potential target groups, i.e., for whom the application of a certain BCT with regard to its functional mechanism might be beneficial. Finally, the empowerment potential is summarized, which can help those practitioners who are interested in the normative implications or potential longevity of a BCT.

**TABLE 2:** Summary of the model and application guide.

Functional mechanisms of BCTs	Definition	Resources targeted	BCT examples in private, public, corporate, and clinical settings	Potential target group	Empowerment potential
Facilitating	External resource provision, i.e., creating or presenting socio-environmental resources to allow new behavior	Changeable external resources, e.g., knowledge, environmental context, social influences	Private: Social support (e.g., self-organized walking groups) Public: Restructuring the physical environment (e.g., public fitness trails) Corporate: Adding objects to the environment (e.g., healthy dishes at the canteen) Clinical: Instruction on how to perform the behavior (e.g., posters)	Individuals who are aware of the benefits of behavior change and motivated, but lack the external resource to implement the new behavior	Direct empowerment
Boosting	Reflective resource build-up, i.e., offering enjoyable tasks that will strengthen internal reflective resources that can support behavior change	Changeable reflective internal resources, e.g., social/professional role and identity, goals, skills, beliefs about capabilities, beliefs about consequences, intentions, behavioral regulation, memory, attention, and decision processes	Private: Self-monitoring of behavior (e.g., through self-awareness/mindfulness courses) Public: Information about health consequences (e.g., interactive exhibitions in public spaces) Corporate: Action planning (e.g., one-to-one session on planning actions to implement or maintain a new behavior) Clinical: Demonstration of the behavior (e.g., cooking course)	Individuals who are aware of the benefits of behavior change and motivated, but lack the capability/ability to implement the new behavior	Self-directed empowerment
Nudging	Affective resource use, i.e., intentionally applying stimuli, cues, or triggers in an environment that activate affective components of decision-making in order to drive behavior	Changeable affective internal resources, e.g., reinforcement, emotions, optimism	Private: Social influence (e.g., a family member serves as a role model) Public: Priming (e.g., arrows on the stairs call for movement *via* the visual pathway) Corporate: Social reward (e.g., point systems/gamification) Clinical: Restructuring the physical environment (e.g., placing healthy dishes in front of unhealthy dishes in canteens)	Individuals who are unaware of the benefits of behavior change or individuals who are aware of the benefits but lack motivation to implement the new behavior	Indirect empowerment

## Limitations and Future Research

The goal of our study was not to provide an exhaustive account of or discuss the wide variety of BCTs in all detail. In fact, the abstraction of BCTs into only three types is rather gross, at the same time providing a first notion of BCTs’ functional mechanisms. We suggest that not all of these dimensions (facilitating, boosting and nudging) are independent of each other, i.e., some BCTs cannot be assigned unilaterally to only one mechanisms. For example, financial incentives for weight loss may be both facilitating and nudging at the same time. The promise of a financial reward for behavior change provides (the expectations of) financial means to buy products that supports the persuasion of the behavioral goal, which might not have been affordable without the expected payoff. At the same time, the announced payoff activates the expectation of a positive affect–potentially for both having lost weight and being paid for the efforts. Despite such overlaps of categories, in our view, these are sufficiently important to merit separate discussion.

All three types of BCTs we have defined are thought to be umbrella terms for a variety of BCTs, which in each category share the same functional mechanism. A more detailed differentiation of functional mechanisms, such as short-term and long-term boosts (Hertwig and Grüne-Yanoff, 2017), or the elaboration of different nudges as in the MINDSPACE taxonomy by Dolan et al. (2012), might be possible. Our goal was to provide a first, general understanding of the functional mechanisms of BCTs.

Our conceptual review is brief and theoretical rather than empirical and exhaustive. The literature we chose to integrate was therefore rather pragmatic and narrative. For example, in order to categorize resources into three types, we did not conduct a systematic search on all theoretical and empirical studies discussing them, but relied on already established frameworks, such as the TDF, which has resulted from extensive work among many behavioral psychologists (Michie et al., 2005; Cane et al., 2012). We believe that the TDF serves the purpose of illustrating the various types of resources necessary for behavior change. In future studies, a more refined assignment of already established resources to the three categories (external, internal reflective and internal affective) is worth elaborating. We also did not provide a full discussion of all dual-process models available in the literature. Instead, our goal was to illustrate the relevance of these models to understand how BCTs work. Finally, we also did not conduct an extensive literature review on the role of motivation and reward proceedings in behavior change. Rather, we relied on a recently established account by Michaelsen and Esch (2021), who discussed the role of motivation and reward proceedings in behavior change in detail. In summary, for this elaboration we relied on existing reviews of the different strands of research, which allowed us to draw an overview of possible aspects important to behavior change. By doing so, we could identify relations which might have been yet overseen due to focus on isolated strands of research which has limited successful developments of BCTs.

Future studies can test the model in at least three ways. First, using neuroscientific methods such as brain imaging to analyze the affective and motivational components involved in various BCTs classified here as Boosting or Nudging techniques. Second, qualitative research could help elaborate how participants of particular intervention types perceive the mechanisms that lead or do not lead them to change their behavior. Third, a systematic review or meta-analysis of intervention studies could help to determine whether the three types of BCTs indicated here are moderators of effectiveness or best used in certain combinations. We also hope for future research to elaborate in more detail on the specific functional mechanisms of BCTs. Especially, future research can dive deeper into the understanding of affective processes involved in nudging interventions, for example, through the investigation of affective states before, during and after decision-making. An understanding of the neurobiological mechanisms involved in all three functional mechanisms (we established here) would also be fruitful to provide even better ground not only for scientific progress but also for future intervention development and implementation in everyday life.

## Conclusion

Health behavior change is among the top recommendations for improving health and strengthening empowerment of patients with lifestyle-related chronic diseases, which constitute the largest part of the disease burden. An array of behavior change techniques (BCTs) have been developed to support behavior change initiation and maintenance. These BCTs often show limited success when they are not informed by theory, leading to a mismatch between the intention of the BCT and patients’ requirements or expectations. Previous studies have identified a number of resources (domains) patients may require to initiate and to maintain health behavior change in order to inform intervention development and implementation. Here, we provided an understanding of how BCTs address these resources, or lacks of resources, i.e., their functional mechanisms.

We extracted the most relevant findings from social psychological theories and incorporated knowledge from behavioral economics and neuroscience. Dual-process models offer powerful illustrations of decision-making and behavior but they have not been adapted to functional mechanisms of BCTs so far. In addition, applying recent understandings of motivation and reward proceedings to the understanding of how BCTs address resources generates a new understanding of the causal but multidirectional relationships between BCTs, behavior and reward mechanisms.

The Behavior Change Resource Model focuses on positive resources, which are, when sufficiently available, likely not only to empower individuals to implement their desired behavior in a joyful way, but also enhance mental health, lead to more productivity and satisfaction over and above improving physical health.

This model is applicable to complex behaviors (such as regular physical exercise) as well as simple behaviors (such as the use of preventive screening services) as it recognizes that health behavior change involves many components and that individuals are diverse not only in terms of their desires and needs but also in terms of personality traits, states, and preferences.

By transferring knowledge across various fields, the present model can inform both researchers aiming at extending the knowledge on human behavior as well as intervention developers. Hence, it can be applied in different contexts–e.g., private, public, corporate, and clinical settings.

## Author Contributions

MM searched the literature, conducted the analysis, and presented a draft of the manuscript. Both authors discussed and refined the results. TE revised the manuscript.

## Conflict of Interest

The authors declare that the research was conducted in the absence of any commercial or financial relationships that could be construed as a potential conflict of interest.

## Publisher’s Note

All claims expressed in this article are solely those of the authors and do not necessarily represent those of their affiliated organizations, or those of the publisher, the editors and the reviewers. Any product that may be evaluated in this article, or claim that may be made by its manufacturer, is not guaranteed or endorsed by the publisher.
